# *Neisseria gonorrhoeae* infects the human endocervix by activating non-muscle myosin II-mediated epithelial exfoliation

**DOI:** 10.1371/journal.ppat.1006269

**Published:** 2017-04-13

**Authors:** Liang-Chun Wang, Qian Yu, Vonetta Edwards, Brian Lin, Jessica Qiu, Jerrold R. Turner, Daniel C. Stein, Wenxia Song

**Affiliations:** 1 Department of Cell Biology & Molecular Genetics, University of Maryland, College Park, Maryland, United States of America; 2 Department of Microbiology and Immunology, University of Maryland School of Medicine, Baltimore, Maryland, United States of America; 3 Departments of Pathology and Medicine (GI), Brigham and Women's Hospital, Harvard Medical School, Boston, Massachusetts, United States of America; Tufts University School of Medicine, UNITED STATES

## Abstract

Colonization and disruption of the epithelium is a major infection mechanism of mucosal pathogens. The epithelium counteracts infection by exfoliating damaged cells while maintaining the mucosal barrier function. The sexually transmitted bacterium *Neisseria gonorrhoeae* (GC) infects the female reproductive tract primarily from the endocervix, causing gonorrhea. However, the mechanism by which GC overcome the mucosal barrier remains elusive. Using a new human tissue model, we demonstrate that GC can penetrate into the human endocervix by inducing the exfoliation of columnar epithelial cells. We found that GC colonization causes endocervical epithelial cells to shed. The shedding results from the disassembly of the apical junctions that seal the epithelial barrier. Apical junction disruption and epithelial exfoliation increase GC penetration into the endocervical epithelium without reducing bacterial adherence to and invasion into epithelial cells. Both epithelial exfoliation and junction disruption require the activation and accumulation of non-muscle myosin II (NMII) at the apical surface and GC adherent sites. GC inoculation activates NMII by elevating the levels of the cytoplasmic Ca^2+^ and NMII regulatory light chain phosphorylation. Piliation of GC promotes, but the expression of a GC opacity-associated protein variant, OpaH that binds to the host surface proteins CEACAMs, inhibits GC-induced NMII activation and reorganization and Ca^2+^ flux. The inhibitory effects of OpaH lead to reductions in junction disruption, epithelial exfoliation, and GC penetration. Therefore, GC phase variation can modulate infection in the human endocervix by manipulating the activity of NMII and epithelial exfoliation.

## Author summary

*Neisseria gonorrhoeae* (GC) infects human genital epithelium causing gonorrhea, a common sexually transmitted infection. Gonorrhea is a critical public health issue due to increased prevalence of antibiotic-resistant strains. Because humans are the only host for GC, a lack of a human infection model has been a major obstacle to our understanding of GC infection. Here we use a human tissue explant model to examine the mechanism by which GC infect the human endocervix, the primary site for GC infection in women. We show that GC penetrate into the human endocervix by activating the actin motor myosin and epithelial shedding. Myosin activation causes the disruption of the endocervical epithelial barrier by inducing apical junction disassembly and epithelial cell shedding, allowing GC penetration into the human endocervical tissue. GC activate myosin by inducing Ca^2+^-dependent phosphorylation of myosin light chain. We further show that GC can enhance and reduce the penetration by expressing pili and the opacity-associated protein that promotes and inhibits myosin activation, respectively. Our study is the first demonstration of GC penetration into the human endocervix. Our results provide new insights into the mechanism by which GC manipulate signaling and cytoskeletal apparatus in epithelial cells to achieve penetrating and non-penetrating infection.

## Introduction

Microbial pathogens establish infection at the mucosal surface by colonization, disruption, and penetration of the epithelium [1]. The epithelium is the first line of the host defense against microbial pathogens, providing a physical barrier and a sensor of invading pathogens [2, 3]. In the female reproductive tract (FRT), this mucosal surface is composed of multilayered non-polarized squamous epithelial cells at the ectocervix and vagina, or monolayered polarized columnar epithelial cells at the endocervix and uterus. Different from multilayered squamous epithelial cells that are held together by adherent junctions, the monolayer epithelium is sealed by the apical junction, which prevents the entry of pathogens through the paracellular space (gate function) and maintains the polarity of the apical and basolateral surfaces (fence function) [4–7]. The apical junction is formed by the integral proteins, claudin, occludin, junctional adhesion molecules, E-cadherin, and the associated proteins zonula occludens-1 (ZO1) and β-catenin [7]. ZO1 and β-catenin link the apical junction to the actin cytoskeleton and signaling networks [7–11]. The actin cytoskeleton and non-muscle myosin II (NMII) form a supporting ring at the apical junction [12–14]. The contraction of the actomyosin ring can transiently open the “gate” of the apical junction, regulating the permeability of the epithelium [15–17]. Over activation of the actomyosin ring can lead to the disassembly of the apical junction by inducing the endocytosis of junctional proteins [18, 19].

As a strategy of protecting the epithelium from pathogens, infected cells with associated microbes are shed [20]. While the exfoliation of multilayered squamous epithelium is mediated by weakening cell-cell adhesion, exfoliation of polarized epithelial monolayers requires collaboration between NMII and apical junctional complexes. Actomyosin and apical junctional proteins are recruited to the plasma membrane of epithelial cells in contact with an exfoliating cell. NMII-generated forces in neighboring cells “squeeze” the exfoliating cell out while apical junctional complexes ensure that the epithelial barrier remains uncompromised [21–25]. How bacteria break the epithelial barrier and escape from epithelial shedding to achieve infection remains elusive.

*Neisseria gonorrhoeae* (GC), a Gram-negative bacterium, infects the mucosal surface of human genital tissues in men and women and causes one of the most common sexually transmitted infections, gonorrhea [26]. In the FRT, the endocervix has been suggested as a primary site for GC to initiate infection that may lead to pelvic inflammatory disease [27, 28]. Previous studies, using epithelial cells, fallopian tube organ culture, and mouse vaginal infection models, have shown that GC can adhere to, invade into, and transmigrate across epithelial cells [29–31]. However, how GC infect the polarized human columnar endocervical epithelial cells has not been well studied. GC major surface molecules, including pili, lipooligosaccharide (LOS), porin, and opacity-associate protein (Opa), function concertedly for infection. Opa has been suggested to be involved in GC adherence to, invasion into, and transmigration across polarized epithelial cells [32–36], as well as GC-GC interaction by binding to LOS [37–39]. Opa, pili and LOS undergo phase variation. This phase variation has been implicated for the capability of GC to infect various locations of the FRT and generate different pathological conditions and complications [32, 40, 41]. Most GC isolated from patients [42] and infected mice are Opa positive [43], underscoring the importance of Opa in infections. Opa has been shown to inhibit GC-induced exfoliation of squamous epithelial cells from the lower genital tract of mice by engaging carcinoembryonic antigen-related cell adhesion molecules (CEACAMs) and activating integrin, which enhances GC colonization [44, 45]. These data indicate that Opa phase variation is a major way for GC to modify their pathogenicity.

GC establishes infection by interacting with various receptors on epithelial cells, such as the binding of Opa to CEACAMs or heparin sulfate proteoglycans (HSPG) [34, 46–48]. These interactions alter signaling cascades in epithelial cells, such as phosphatidylinositol 3-kinase, phospholipase C, and Ca^2+^ flux. The signaling leads to actin reorganization, which can drive microvillus elongation and the subsequent engulfment of GC [49, 50]. We have shown that GC-induced transactivation of epidermal growth factor receptor (EGFR) is critical for the optimal level of GC invasion into non-polarized epithelial cells and transmigration across polarized epithelial cells [51, 52]. GC interaction with polarized epithelial cells weakens the apical junction by inducing the disassociation of ZO1 and β-catenin from the junctional complex, consequently facilitating GC transmigration [51]. Our recently published studies found a surprising role for Opa in inhibiting GC transmigration across polarized epithelial cells [38]. How GC manipulate columnar endocervical epithelial cells through Opa for infection is unknown. A major obstacle against addressing this question has been a lack of infection models that mimic all aspects of human infection.

In this study, we established a new *ex vivo* infection model, human endocervical tissue explants. Using this model and polarized epithelial cells, as well as isogenic strains of GC expressing invariable Opa, we revealed the mechanistic links between GC infectivity, GC-induced exfoliation, apical junction disassembly, and signaling in polarized columnar endocervical epithelial cells, and novel roles of Opa phase variation in these events. GC induce the exfoliation of polarized endocervical epithelial cells by disrupting the apical junction. Opposite to GC-induced shedding of squamous epithelial cells, the exfoliation of columnar epithelial cells does not reduce GC adherence and invasion; instead, it increases GC penetration into the subepithelium. Both GC-induced epithelial exfoliation and apical junction weakening require Ca^2+^-dependent redistribution of active NMII. The expression of CEACAM-binding OpaH but not HSPG-binding OpaC inhibits GC-induced exfoliation and junctional disruption by interfering with NMII activation and reorganization as well as Ca^2+^ flux, while GC piliation promotes these events. Our results suggest that GC modify the exfoliation process for infection by activating Ca^+^ flux and NMII redistribution in endocervical epithelial cells and change the magnitude of this process through regulating the levels of NMII activation and redistribution by Opa and pili phase variation.

## Results

### GC-induced epithelial exfoliation from human endocervical tissue explants and polarized monolayers is inhibited by Opa expression

**Fig 1:**
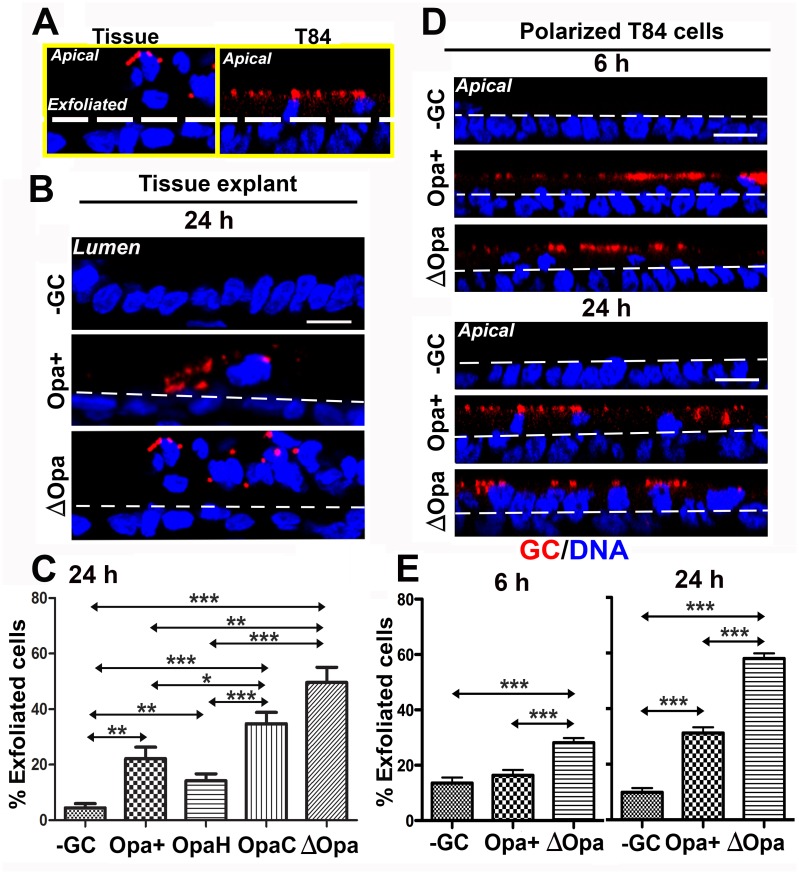
GC induce exfoliation of polarized epithelial cells from human endocervical tissue explants and T84 monolayers while the expression of CEACAM-binding Opa suppresses the exfoliation. Human endocervical tissue pieces (A-C) and polarized T84 monolayers (A, D, and E) were apically incubated with piliated MS11Opa+, ΔOpa, OpaH, or OpaC at a MOI of ~10 for 6 or 24 h at 37°C, with unassociated GC washed off at 6 and 12 h. Cells were fixed, stained for DNA and GC, and analyzed using 3D-CFM. Shown are representative images that intercept both the apical and basolateral surfaces (Scale bar, 10 μm) (A, B, and D). Based on cell nuclear staining, the average percentage (±SD) of exfoliated epithelial cells was determined by counting the number of epithelial cells localizing above the endocervical epithelium (A-C) and T84 monolayers (A, D, and E), indicated by white dash lines, versus the total number of epithelial cells. Shown are the results from >15 randomly selected fields (>50 cells) from three independent experiments or cervixes of two to three human subjects. ***p ≤0.001; **p ≤ 0.01; *p≤0.05.

We utilized human endocervical tissue explants and the polarized human colonic epithelial cell line T84 to determine whether GC-infected polarized epithelial cells undergo exfoliation. Tissue explants that were cultured with the mucosal side up and T84 cells that were polarized on transwells were inoculated apically with a GC strain, MS11 that express phase variable Opa and pili (MS11Pil+Opa+) at a MOI of ~10 for 6 or 24 h. Thin sections of cryo-preserved endocervical tissues and T84 cells were stained with a DNA dye and GC-specific polyclonal antibodies and analyzed using and three-dimensional confocal fluorescence microscopy (3D-CFM). Images showing both the mucosal and subepithelial sides of the endocervix and T84 monolayers were analyzed. Epithelial cells at the top of the endocervical epithelium of tissue explants or T84 monolayers, indicated by white lines, were counted as exfoliating cells (Fig 1A) and quantified as the percentage of total epithelial cells. After 24 h incubation, the exfoliation of GC-inoculated epithelial cells was significantly increased in both the endocervical epithelium (Fig 1B and 1C) and the T84 monolayer (Fig 1D and 1E), compared to uninfected controls. This indicates that polarized T84 monolayers behave similarly to the endocervical epithelium upon GC infection. There was no significant increase in the percentage of GC-inoculated epithelial cells exfoliated from T84 monolayer after 6-h inoculation, compared to uninfected cells (Fig 1E, left panel).

To determine if Opa has a role in the exfoliation of columnar epithelial cells, we inoculated endocervical tissue explants and polarized epithelial cells with MS11Pil+ΔOpa, a GC strain where all 11 *opa* genes were deleted [39]. MS11Pil+ΔOpa increased the percentage of epithelial exfoliation from 32.3% to 66.3% in tissue explants (Fig 1B and 1C) and from 31.2% to 55.8% in T84 monolayers (Fig 1D and 1E). Even at 6 h, MS11Pil+ΔOpa-infected T84 cells exfoliated significantly more than the uninfected control (Fig 1D and 1E). To determine whether different Opa variants have similar effects on the exfoliation of endocervical epithelial cells, we utilized MS11Pil+ΔOpa strains that express invariant OpaH (binding to CEACAMs) or OpaC (binding to HSPG). We found that the exfoliation level of MS11Pil+OpaH-inoculated endocervical tissue explants was as low as that of MS11Pil+Opa+ infected explants, while the exfoliation level of MS11Pil+OpaC-inoculated explants was as high as that of MS11Pil+ΔOpa-infected explants (Fig 1C). These results indicate that GC induces the epithelial exfoliation from the endocervix and cell line-formed polarized monolayers, and the expression of CEACAM-binding OpaH but not HSPG-binding OpaC inhibits the exfoliation. The similar inhibitory effect of MS11Opa+ and MS11OpaH on epithelial exfoliation suggests that MS11Opa+ expresses primarily CEACAM-binding Opa proteins.

### GC-induced epithelial exfoliation depends on the activation of non-muscle myosin II and Ca^2+^ flux

**Fig 2:**
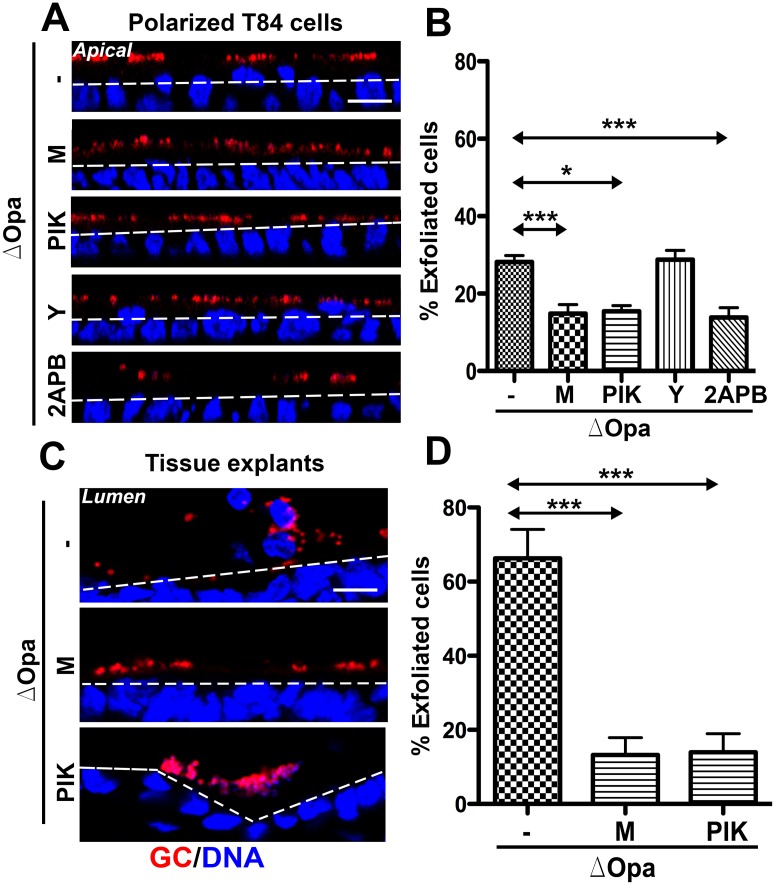
GC induce exfoliation of polarized epithelial cells requires the activation of Ca^2+^ flux and Non-muscle Myosin II (NMII) by Myosin Light Chain Kinase (MLCK). Polarized T84 cells (A and B) and human endocervical tissue explants (C and D) were untreated or pre-treated with the ROCK inhibitor Y27632 (Y, 10 μM) or the MLCK inhibitors ML-7 (M, 10 μM) or PIK (100 μM) for 1 h and apically incubated with piliated MS11Opa+ or ΔOpa for 6 h or 24 h in the presence or absence of inhibitors. Cells were fixed, stained for DNA and GC, and analyzed using 3D-CFM. Shown are representative images (Scale bar, 10 μm) (A and C). The average percentages (±SD) of exfoliated cells were determined as Fig 1 from >15 randomly selected fields (>50 cells) of three independent experiments or cervixes of two to three human subjects. ***p ≤0.001; **p ≤ 0.01; *p≤0.05.

To determine if GC-induced exfoliation of endocervical epithelial cells depends on NMII, we inhibited the activation of NMII using inhibitors specific for Rho-associated kinase (ROCK), Y27632, and myosin light chain kinase (MLCK), ML-7 and PIK. Polarized T84 cells and human endocervical tissue explants were treated with individual inhibitors for 1 h before and during incubation with GC. We found that both the small chemical inhibitor (ML-7) [53] and the catalytic site-targeted peptide inhibitor (PIK) [54] of MLCK reduced the exfoliation of MS11Pil+ΔOpa-infected (Fig 2A and 2B) but not MS11Pil+Opa+-infected epithelial cells from T84 monolayers (S1A and S1B Fig). In contrast, treatment with the ROCK inhibitor did not significantly change the percentage of epithelial exfoliation, no matter if epithelial cells were infected with MS11Pil+Opa+ (S1A and S1B Fig) or MS11Pil+ΔOpa (Fig 2A and 2B). Importantly, the treatment of MLCK inhibitor, ML-7 or PIK, also decreased the epithelial exfoliation of human endocervical tissue explants to the basal level no matter if it was based on the total number of epithelial cells (Fig 2C and 2D) or GC-associated epithelial cells (S1C Fig).

As MLCK activation requires Ca^2+^-bound calmodulin [55, 56] and the MLCK inhibitor PIK blocks the calmodulin-binding site in MLCK [54], we investigated if GC-induced exfoliation of polarized epithelial cells depends on Ca^2+^ flux. We utilized 2APB, an inhibitor that blocks Ca^2+^ release from intracellular stores [57, 58]. Treatment with 2APB also reduced the exfoliation of polarized T84 cells to the level similar to ML-7 and PIK (Fig 2A and 2B). As controls, we treated polarized T84 cells with the inhibitors alone, and found that ML-7, PIK, and 2APB did not affect epithelial exfoliation, but the NMII motor inhibitor blebbistatin increased epithelial exfoliation without GC inoculation (S2 Fig). These results suggest that GC induce exfoliation of polarized epithelial cells via Ca^2+^- and MLCK- but not ROCK-dependent activation of NMII.

### Expression of CEACAM-binding Opa inhibits GC penetration into polarized epithelial cells without reducing GC adherence

**Fig 3:**
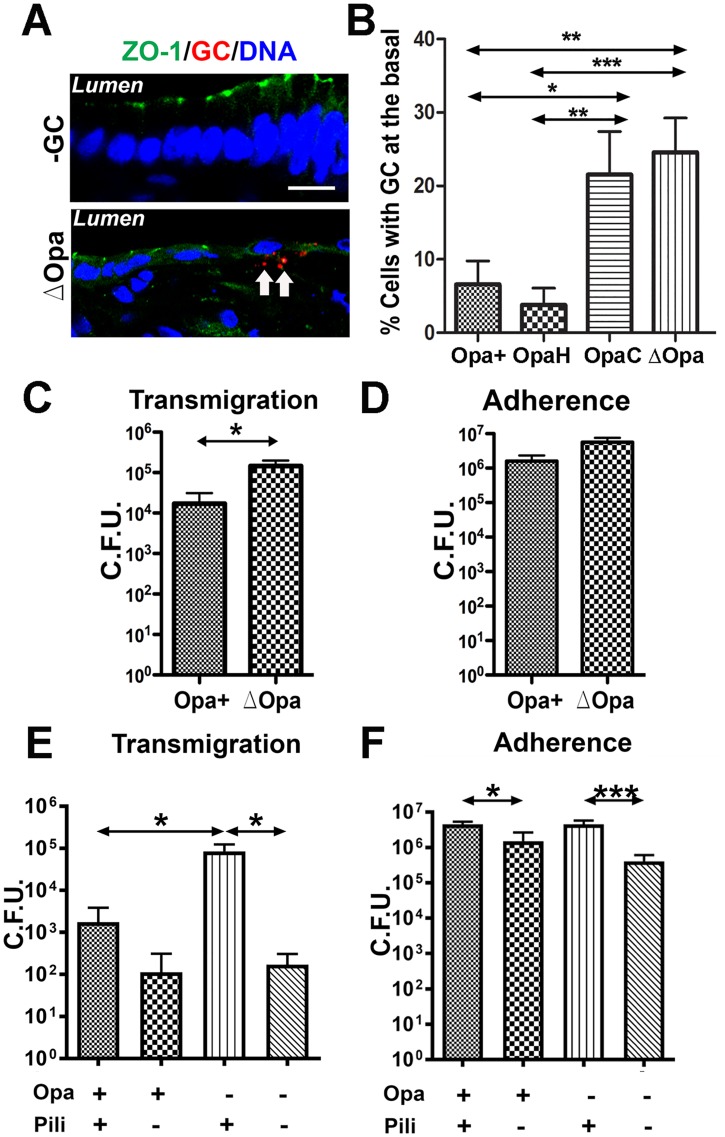
Effects of Opa and pili on GC adherence to and transmigration across polarized epithelial cells. (A-B) Human endocervical tissue explants were incubated with piliated MS11Opa+, ΔOpa, OpaH, or OpaC for 24 h, stained for ZO-1, nuclei and GC, and analyzed by 3D-CFM (A). GC subepithelial penetration (arrows) was quantified using 3D-CFM images as the percentage of epithelial cells with basal GC staining among the total number of GC-associated epithelial cells (B). Shown are the average values (±SD) of >50 epithelial cells of endocervical tissue explants from two to three human subjects. (C and D) Polarized HEC-1-B cells were apically incubated with piliated MS11Opa+ or ΔOpa. The basal medium was collected after 6 h to determine transmigrated GC (C). The epithelial cells were lysed after 3 h incubation and washing to quantify adherent GC (D). (E-F) Polarized T84 cells were apically incubated with piliated or non-piliated MS11Opa+ or ΔOpa for 6 or 3 h, and the numbers of transmigrated (E) and adherent GC (F) were determined as described above. Shown are the means (±SD) of >6 transwells from 4–6 independent experiments. ***p ≤0.001; **p ≤ 0.01; *p≤0.05.

We have previously shown that GC can transmigrate across polarized epithelial cells, and Opa expression inhibits the transmigration [38]. To determine whether such transmigration occurs in the endocervical epithelium and which Opa variant inhibits GC transmigration, we utilized the tissue explants and GC strains expressing single invariable Opa. After incubating with piliated GC for 24 h, we examined GC transmigration across the endocervical epithelium by quantifying the percentage of GC-associated endocervical epithelial cells with GC staining in the basal side (Fig 3A). In infected endocervical tissue explants, the percentages of epithelial cells associated with penetrated MS11ΔOpa and MS11OpaC were significantly higher than those with penetrated MS11Opa+ and MS11OpaH (Fig 3B). However, there was no significant difference between the percentages of epithelial cells with penetrated MS11ΔOpa and MS11OpaC and between those with penetrated MS11Opa+ and MS11OpaH (Fig 3B). These results indicate that GC can penetrate into the subepithelium of the human endocervix in the tissue explant model, and the expression of CEACAM-binding OpaH, which reduces GC-induced epithelial exfoliation, but not HSPG-binding OpaC, which does not affect the exfoliation, inhibits GC penetration.

We next asked whether Opa-mediated inhibition of penetration is related to the efficiency of GC adherence using polarized human endometrial epithelial cells, HEC-1-B (Fig 3C and 3D), and T84 monolayers (Fig 3E and 3F). Similar to what we observed in the endocervical tissue, the numbers of MS11Pil+ΔOpa transmigrating across polarized HEC-1-B (Fig 3C) and T84 monolayers (Fig 3E) were significantly higher than those of MS11Pil+Opa+. However, the expression of Opa had no significant effect on GC adherence to the apical surface of HEC-1-B (Fig 3D) and T84 cells (Fig 3F).

Pili have been shown to be involved in GC transmigration across polarized epithelial cells [59, 60]. To examine the relationship between pili and Opa, we compare the transmigration and adherence efficiencies of piliated and non-piliated MS11Opa+ and MS11ΔOpa in polarized T84 cells (Fig 3E and 3F). We found that the numbers of non-piliated MS11Opa+ and MS11ΔOpa that adhered to and transmigrated across epithelial monolayers were significantly lower than their piliated strains. However, Opa expression only reduced the transmigration of piliated but not non-piliated GC (Fig 3E). These results suggest that pili and Opa play opposing roles in GC transmigration, with pili promote GC transmigration, probably by enhancing adherence, and Opa inhibiting GC transmigration without affecting GC adherence.

### Ca^2+^ flux and NMII activation are required for GC penetration into but not GC adherence to polarized epithelial cells

**Fig 4:**
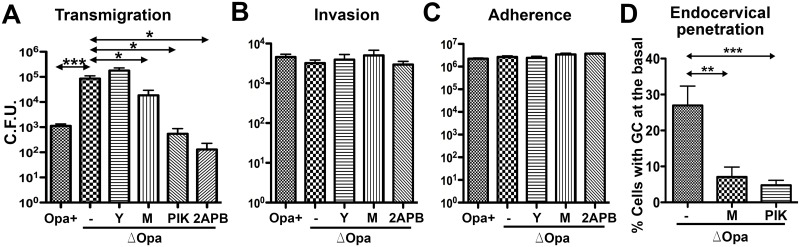
Inhibition of Ca^2+^ signal and NMII activation reduces GC penetration into the epithelium but not GC adherence and invasion. (A-C) Polarized T84 cells were untreated or pre-treated with the ROCK inhibitor Y27632 (Y), the MLCK inhibitors ML-7 (M) and PIK, or the intracellular Ca^2+^ release inhibitor 2APB (10 μM), and apically incubated with piliated MS11Opa+ or ΔOpa for 6 h in the presence or absence of inhibitors. The basal medium was collected to determine transmigrated GC (A). Invaded (B) and adhered GC (C) were quantified by the gentamicin resistance assay. (D) Human endocervical tissue explants were incubated with piliated MS11ΔOpa for 24 h in the presence or absence of ML-7 and PIK. GC subepithelial penetration was quantified using 3D-CFM images as the percentage of epithelial cells with basal GC staining among the total number of GC-associated epithelial cells. Shown are the average values (±SD) of >50 epithelial cells of endocervical tissue explants from two to three human subjects. ***p ≤0.001; **p ≤ 0.01; *p≤0.05.

The inhibitory effects of CEACAM-binding Opa on both GC-induced epithelial exfoliation and GC penetration in the endocervical tissue explants implicate a relationship between these two events. To investigate this relationship, we determined whether inhibiting GC-induced exfoliation would affect the ability of GC to adhere to, invade into, and transmigrate across polarized epithelial cells. Inhibition of GC-induced exfoliation by the Ca^2+^ (2APB) and MLCK inhibitors (ML-7 and PIK) significantly reduced the transmigration of MS11Pil+ΔOpa across the polarized T84 monolayer (Fig 4A). However, none of these inhibitors had any significant effect on the adherence and invasion of MS11Pil+ΔOpa (Fig 4B and 4C). The ROCK inhibitor that did not affect GC-induced exfoliation also had no impact on GC adherence, invasion and transmigration (Fig 4A–4C). Treatment with the inhibitors alone did not significantly affect the barrier function of the epithelium and GC growth except that treatment of PIK longer than 6 h reduced the overall yield of gonococci to one-half (S3 Fig). Similar to the results obtained from polarized T84 cells, treatment with either ML-7 or PIK decreased the percentage of epithelial cells with basally associated GC among the total GC-associated epithelial cells from 27% to 7.1% (Fig 4D), significantly inhibiting GC penetration into the endocervical epithelium. Our results suggest that Ca^2+^ flux and the activation of NMII by MLCK in polarized epithelial cells, which are required for GC-induced epithelial exfoliation, also are critical for GC transmigration across and penetration into the human endocervical epithelium, but not for GC adherence and invasion.

### GC-induced junction disassembly requires NMII activation and Ca^2+^ flux but is suppressed by Opa

**Fig 5:**
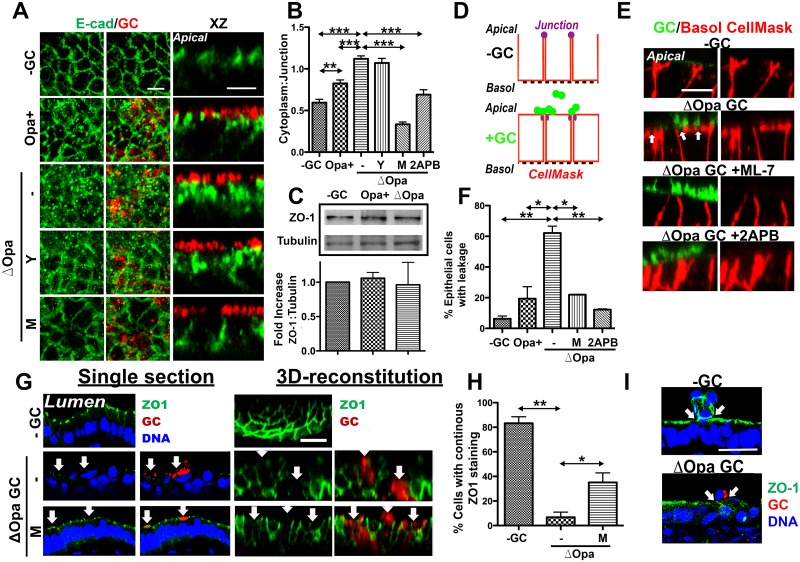
GC induce apical junction disruption in polarized epithelial cells and endocervical tissue explants in a Ca^2+^- and MLCK-dependent manner. (A and B) Effects of NMII kinase and Ca^2+^ inhibitors on the distribution of E-cadherin. Polarized T84 cells were untreated or pre-treated with NMII kinase inhibitors, Y27632 (Y) and ML-7 (M), or a Ca^2+^ inhibitor, 2APB, and then apically incubated with piliated MS11Opa+ or ΔOpa for 6 h in the presence or absence of inhibitors. (A) Cells were fixed, stained for E-cadherin (E-Cad) and GC, and analyzed using 3D-CFM. (B) The average fluorescence intensity ratios (FIR) (±SD) of E-Cad staining at the cytoplasmic to the cell-cell junctional region was determined from >50 cells of three individual experiments using the NIH ImageJ software. (C) The expression levels of ZO1 in MS11Pil+Opa+- or ΔOpa-infected T84 cells were compared using Western blotting and quantified by the average fold of increases (±SD) in the ratio of ZO1 to tubulin in cell lysates from three independent experiments. (D-F) Effects of MLCK and Ca^2+^ inhibitors on the membrane lateral movement over the apical junction. (D) Polarized T84 cells treated with inhibitors as above were apically inoculated with fluorescently labeled piliated GC for 4 h and basolaterally stained with CellMask for 15 min. (E) Time lapse xz images were acquired using CFM. (F) The average percentage (±SD) of cells showing the basolaterally stained dye moving over to the apical surface was determined from >50 randomly selected cells of three independent experiments. Scale bar, 5 μm. (G-H) Effects of GC and the MLCK inhibitor ML-7 on the ZO1 distribution in human endocervical tissue explants. The tissue explants were untreated or pre-treated with ML-7 (M) for 1 h and incubated with MS11Pil+ΔOpa for 24 h in the absence or presence of ML-7. Tissues were stained for ZO1, DNA, and GC. (G) Shown are representative CFM (left panels, arrows to GC) and 3D reconstituted images (right panels) (Bar, 10 μm). (H) The average percentages (±SD) of GC-associated cells showing continuous ZO1 staining at the apical region were determined from >15 randomly selected fields (>50 cells) from cervixes of two to three human subjects. ***p ≤0.001; **p ≤ 0.01; *p≤0.05. (I) Representative CFM images of ZO1 distribution in exfoliating and surrounding epithelial cells (arrows) of cervical tissues from 4 human subjects.

The linkage between the efficiency of GC penetration into the epithelium with GC-induced epithelial exfoliation and apical junction disruption shown here and previously [51] implicate GC-induced junction disruption as an underlying cause of epithelial exfoliation. To test this hypothesis, we determined whether Opa expression and the MLCK and Ca^2+^ inhibitors, which all inhibited GC-induced epithelial exfoliation, also prevent GC from disrupting the apical junction. The structural integrity of the apical junction was evaluated by analyzing the distribution of E-cadherin using immunofluorescence (IFM) and 3D-CFM and quantifying the fluorescence intensity ratio (FIR) of E-cadherin at the cytoplasm to that at the apical junction. In polarized T84 cells that were not inoculated with GC, E-cadherin staining was primarily localized at the apical junction (Fig 5A, top panels). Incubation with GC changed the continuous E-cadherin staining at the apical junction into puncta in the cytoplasm, indicating endocytosis of E-cadherin (Fig 5A). This led to a significant increase in the cytoplasm: junction FIR of E-cadherin in both MS11Pil+Opa+ and MS11Pil+ΔOpa-inoculated epithelial cells, compared to non-inoculated controls (Fig 5B). In particular, the magnitude of the increase in the FIR was significantly greater in MS11Pil+ΔOpa-infected than MS11Pil+Opa+-infected epithelial cells (Fig 5B). Our Western blot analysis did not find any significant changes in the protein level of the apical junctional protein ZO1 between epithelial cells inoculated with piliated MS11Opa+, MS11ΔOpa, and no GC (Fig 5C). These results suggest that Opa expression suppresses GC-induced apical junction disassembly by inhibiting E-cadherin endocytosis.

We used inhibitors to determine the role of NMII and Ca^2+^ flux in GC-induced junction disassembly. Treatment with the MLCK inhibitor ML-7 and the Ca^2+^ inhibitor 2APB, but not the ROCK inhibitor Y27632, decreased the punctate staining of E-cadherin in the cytoplasm and the cytoplasm: junction FIR of E-cadherin to or below the control level in epithelial cells without GC inoculation (Fig 5A and 5B). Thus, Ca^2+^/MLCK inhibitors suppress GC-induced junction disassembly.

Our previous studies show that GC-induced junctional disassembly leads to a significant increase in the lateral diffusion between the apical and basolateral membrane but not in the permeability of epithelial monolayers [51]. To determine whether Opa, MLCK and Ca^2+^ flux are involved in this functional alteration of the apical junction, we stained the basolateral surface of polarized T84 epithelial cells exclusively with CellMask dye for 15 min, after apical incubation with fluorescently labeled piliated GC for 6 h. The appearance of basolaterally stained CellMask dye in the apical membrane indicates a decrease in the fence function of the apical junction (Fig 5D). In control cells where no GC were added, <10% of the cells showed the CellMask staining at the apical surface. The percentage of cells with basolaterally labeled CellMask reaching the apical surface increased to 19.4% when MS11Pil+Opa+ was inoculated and to 62.2% when MS11Pil+ΔOpa was inoculated (Fig 5E and 5F). These results indicate that while both Opa+ and ΔOpa GC decrease the fence function of the apical junction, MS11Pil+ΔOpa caused a greater reduction than MS11Pil+Opa+. Moreover, the treatment with the MLCK inhibitor ML-7 or the Ca^2+^ inhibitor 2APB significantly lowered the percentage of epithelial cells with the CellMark staining leaked to the apical surface (Fig 5E and 5F), thereby inhibiting the GC-induced fence function reduction.

We determined if MS11Pil+ΔOpa can induce the disruption of the apical junction in human endocervical tissue. Sections of uninfected and infected tissue explants were stained for the junctional protein ZO1, GC, and DNA and analyzed by 3D-CFM (Fig 5G). We quantified junction disruption by determining the percentage of GC-associated epithelial cells that lost continuous apical staining of ZO1, using 3D reconstituted confocal images (Fig 5G, right panels). After a 24-h incubation with MS11Pil+ΔOpa, ZO1 staining at the apical junction of GC-associated epithelial cells appeared to be reduced (Fig 5G, left panels), and 93.2% of GC-associated epithelial cells showed defective ZO1 staining (Fig 5G, right panels, arrows), compared to 16.7% of uninfected cells (Fig 5H). In contrast to the recruitment of ZO1 to epithelial cells neighboring exfoliating cells in uninfected monolayers, no accumulation of ZO1 staining was observed around GC-infected exfoliating cells (Fig 5I, arrows). Furthermore, GC inoculation significantly changed the morphology of endocervical epithelial cells, with the cells losing their tall and columnar shape (Fig 5G, left panels). Treatment with the MLCK inhibitor ML-7 restored both the morphology (Fig 5G left panels) and apical distribution of ZO1 (Fig 5H). These data confirm the ability of GC to compromise the apical junction of the endocervical epithelial cells in a NMII-dependent manner in the human tissue explants.

These results together show that both Opa expression and Ca^2+^/MLCK inhibitors suppress GC-induced disruption of the apical junction, indicating that similar to GC-induced epithelial exfoliation, Ca^2+^ signal and MLCK-mediated NMII activation are required for GC-induced apical junction disruption while Opa expression inhibits the junction disruption.

### Pili induce and Opa suppresses the redistribution of active non-muscle myosin II in polarized and endocervical epithelial cells

**Fig 6:**
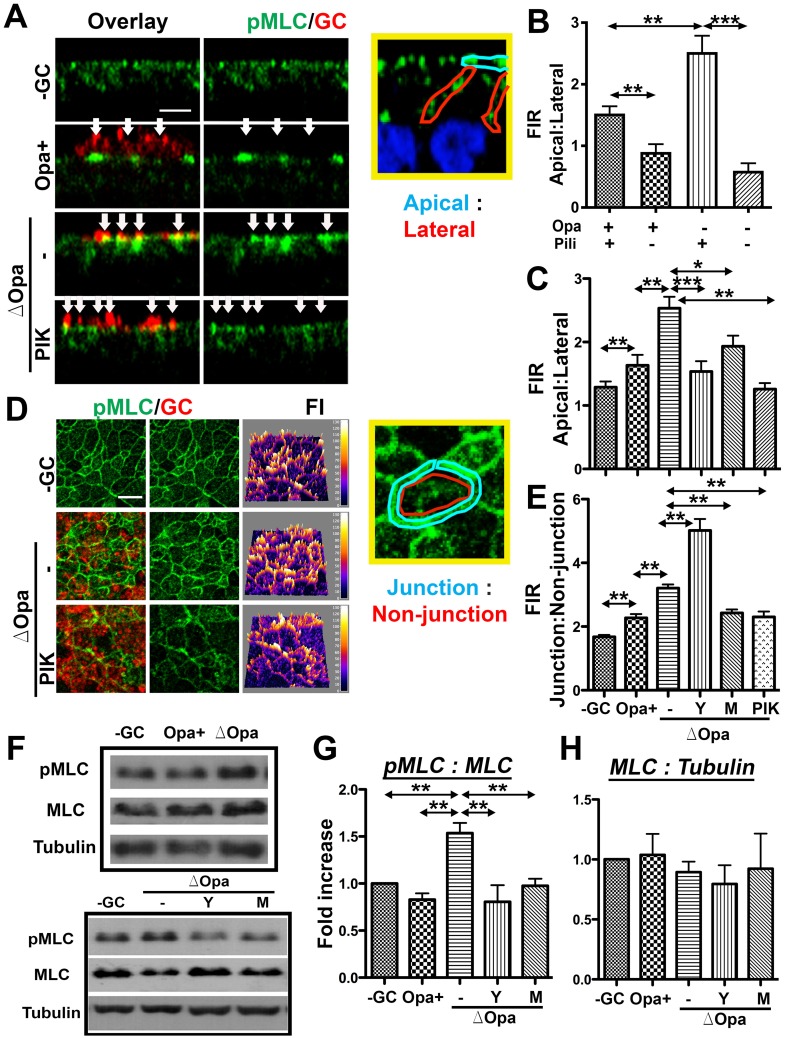
GC interaction increases the levels of phosphorylated MLC at the apical junction and apical membrane. (A-E) Polarized T84 cells were untreated or pre-treated with the ROCK inhibitor Y27632 (Y) and the MLCK inhibitor ML-7 (M) and PIK, and apically incubated with piliated and non-piliated MS11Opa+ and ΔOpa for 6 h in the presence or absence of inhibitors. Cells were stained for phosphorylated MLC (pMLC) and GC and analyzed using 3D-CFM. FIRs of pMLC at the apical to lateral region (A-C) and at the junctional to non-junctional region (D and E) were determined. Shown are representative xz (A) and xy images at the apical junctional location (D), FI maps (D, right panels), and the average FIR (±SD) (B, C, and E) of >50 cells from three independent experiments. Arrows indicate GC. Bar, 5 μm. (F-H) Polarized T84 cells were treated with inhibitors and incubated with piliated GC as above. Cells were then lysed and analyzed by Western blot probing for MLC, pMLC and β-tubulin. The blot was quantified by Phosphorimager. Shown are representative blots (F) and the average fold of increase (±SD) in pMLC:MLC (G) and MLC:tubulin (H) ratios over no GC control from three independent experiments. ***p ≤0.001; **p ≤ 0.01; *p≤0.05.

Our finding of that GC induce both epithelial exfoliation and apical junction disassembly in a NMII-dependent manner suggests that GC regulate the activity of NMII in polarized epithelial cells. We examined the cellular distribution of active NMII after 6-h incubation with GC, using antibody specific for phosphorylated myosin light chain (pMLC) and 3D-CFM. In uninfected polarized T84 (Fig 6A) and HEC-1-B cells (S4A Fig), pMLC was primarily localized at the apical junction. The polarized distribution of pMLC at the apical surface was quantified by the FIR of pMLC at the apical to the lateral (Apical: Lateral) membrane areas in individual cells using CFM images scanning across the apical and basolateral surfaces (Fig 6A–6C). The polarized distribution pMLC at the apical junction was quantified by the FIR of pMLC at the junction to non-junction (Junction: Non-junction) areas of the apical region using CFM images scanning through the apical junction (Fig 6D and 6E). The apical inoculation of piliated MS11Opa+ and MS11ΔOpa caused significant increases in apical: lateral FIR in both polarized T84 (Fig 6A–6C) and HEC-1-B cells (S4 Fig), compared to the no GC control. There were also significant increases in the junction: non-junction FIR in infected polarized T84 cells, compared to non-infected cells (Fig 6D and 6E). Moreover, both the apical: lateral and junction: non-junction FIRs were significantly higher in MS11Pil+ΔOpa-infected than those in MS11Pil+Opa+-infected T84 cells (Fig 6C and 6E), but this difference was not detected in HEC-1-B cells that do not express CEACAMs [61] (S4 Fig). In contrast, the apical: lateral FIR in epithelial cells infected by non-piliated MS11, no matter if GC expressed Opa or no, were all significantly reduced to a similar level, compared to those infected by piliated MS11 (Fig 6B). We further noticed that NMII at the apical surface appeared to accumulate at GC adherent sites (Fig 6A, middle panels, arrows).

To determine if GC inoculation changes the activation level of NMII, we quantified the amount of pMLC and MLC by Western blot. Polarized T84 cells were incubated with or without piliated MS11Opa+ or MS11ΔOpa apically for 6 h before lysis and Western blot analysis. The antibody staining density ratios of pMLC to MLC in MS11Pil+ΔOpa- but not MS11Pil+Opa+-inoculated cells were significantly higher than that in uninoculated epithelial cells (Fig 6F, top panels, and Fig 6G). However, GC inoculation did not significantly change the staining density ratio of MLC to tubulin (Fig 6H). Thus, MS11Pil+ΔOpa, but not MS11Pil+Opa+, increases the activation level of NMII.

**Fig 7:**
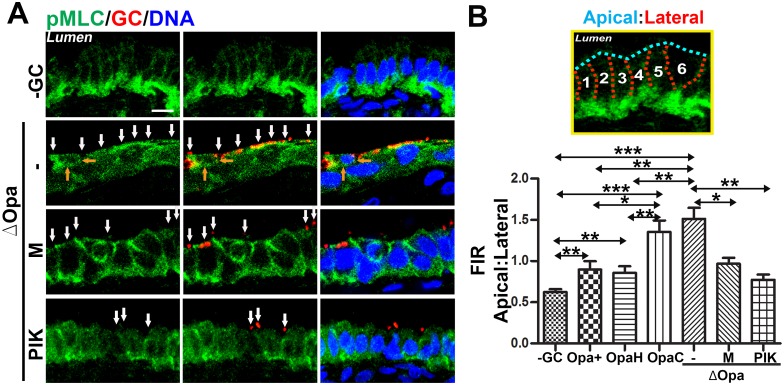
The redistribution of active NMII in GC-infected endocervical epithelial cells of human tissue explants. Human endocervical tissue explants were untreated or pre-treated with the MLCK inhibitor ML-7 (M) or PIK and incubated with piliated MS11Opa+, ΔOpa, OpaH, or OpaC in the presence or absence of the inhibitor for 24 h. Unassociated GC were washed off at 6 and 12 h. The tissue was cryopreserved, sectioned, stained for GC, pMLC, and DNA, and analyzed using 3D-CFM. Shown are representative images (A; white arrows, GC; orange arrows, contacts between an exfoliating cell and its neighboring cells; Bar, 10 μm) and the average FIR (±SD) of pMLC at the apical to lateral region (B) from >40 epithelial cells of cervixes from two human subjects. ***p ≤0.001; **p ≤ 0.01; *p≤0.05.

To explore the possibility of that GC-induced NMII redistribution occurs *in vivo* and the role of Opa phase variation, we incubated human endocervical tissue explants with piliated MS11Opa+, ΔOpa, OpaH, or OpaC for 24 h. Cryo-sections of the endocervical tissue were stained for pMLC, GC and nuclei. In addition to its apical junction localization, pMLC was concentrated at the basal surface of the endocervical epithelial cells contacting with the basal membrane (Fig 7A, upper panels). When incubated with MS11Pil+Opa+, there was a redistribution of pMLC from the basal to apical surface, resulting in a significant higher apical: lateral FIR in GC-inoculated tissue explants than that in no GC control (Fig 7). Inoculation of MS11Pil+ΔOpa further increased the apical: later FIR of pMLC, similar to what we observed in polarized T84 (Figs 7B and 6A–6C). Expression of OpaH, but not OpaC, in MS11Pil+ΔOpa reduced the apical: lateral FIR back to the level in MS11Pil+Opa+-infected cells (Fig 7B). Furthermore, pMLC at the apical surface of the endocervical epithelial cells also concentrated at GC adherent sites (Fig 7A, second row, white arrows), but not at the membrane of cells neighboring exfoliating cells (Fig 7A, second row, orange arrows). These observations confirm that GC increase the relative amount of activated NMII at the apical surface of the endocervical epithelial cells in human tissue explants. Our results from both human endocervical tissue explants and polarized epithelial cell lines suggest that GC interactions via pili cause an accumulation of activated NMII at GC adherent sites and the apical membrane of columnar epithelial cells, and the expression of CEACAM-binding Opa suppresses the activation and redistribution of NMII.

### Differential roles of myosin light chain kinase and Rho-associated protein kinase in GC-induced NMII activation and redistribution

The activation of NMII is mediated by the phosphorylation of MLC by MLCK downstream of Ca^2+^-activated calmodulin [62–65] and/or by ROCK downstream of Rho GTPases [62, 66]. Our findings that GC-induced epithelial exfoliation and apical junctional disruption, as well as GC transmigration, are inhibited by the MLCK and Ca^2+^ but not ROCK inhibitors suggest that MLCK mediates the activation and redistribution of NMII triggered by GC. We determined the effects of the MLCK and ROCK inhibitors on GC-induced MLC redistribution and phosphorylation using 3D-CFM and Western blot. Our 3D-CFM analysis found that treatment with the MLCK inhibitor ML-7 or PIK significantly reduced both the apical: lateral and junction: non-junction FIRs of pMLC in GC-infected epithelial cells (Fig 6A and 6C–6E), as well as the accumulation of pMLC at GC adherent sites (Fig 6A, bottom panels, arrows). However, treatment with the ROCK inhibitor Y27632 further increased the junction: non-junction FIR of pMLC in MS11Pil+ΔOpa-inoculated epithelial cells, while having similar inhibitory effects as the MLCK inhibitors on the apical: lateral FIR of pMLC (Fig 6A and 6C–6E). Our Western blot analysis showed that treatment with either the MLCK or the ROCK inhibitor reduced the pMLC: MLC but not the MLC: tubulin density ration in MS11Pil+ΔOpa-inoculated epithelial cells to basal levels (Fig 6F–6G). Moreover, the MLCK inhibitors ML-7 and PIK significantly reduced the apical: lateral FIR of pMLC (Fig 7) and pMLC accumulation at GC adherent sites (Fig 7A, white arrows) in MS11Pil+ΔOpa-inoculated endocervical tissue explants. These results suggest that both MLCK and ROCK are involved in the activation of MLC phosphorylation induced by MS11Pil+ΔOpa, but MLCK and ROCK distinctly regulate the subcellular location of active NMII with MLCK promoting and ROCK inhibiting the accumulation of active NMII to the apical junction.

### GC inoculation induces Ca^2+^-dependent redistribution of active NMII in polarized epithelial cells

**Fig 8:**
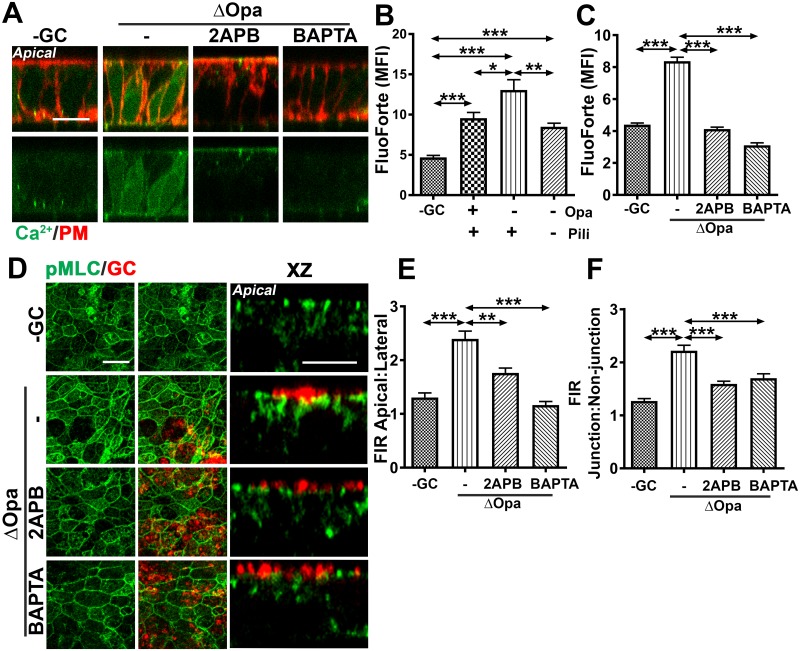
GC-induced redistribution of active NMII depends on Ca^2+^ flux. (A-C) Polarized T84 cells were incubated apically with or without piliated or non-piliated MS11Opa+ or ΔOpa in the absence or presence of Ca^2+^ inhibitors, 2APB (10 μM) or BAPTA (50 μM), for 4 h. Cells were incubated with the Ca^2+^ indicator FluoForte and the membrane dye CellMask and analyzed using 3D-CFM. Shown are representative xz images (A) (Bar, 5 μm) and the average MFI (±SD) of FluoForte in the cytoplasmic region (B-C) of >50 cells from three independent experiments. (D-F) Polarized T84 cells were untreated or pre-treated with the Ca^2+^ inhibitors for 1 h and then apically incubated with piliated MS11Opa+ or ΔOpa for 6 h in the presence or absence of inhibitors. Cells were stained for pMLC and GC and analyzed by 3D-CFM. Shown are representative xy (D, left panels) and xz (D, right panels) images (Bar, 5 μm) and the average pMLC FIRs (±SD) of apical: lateral (E) and junction: non-junction (F) generated from >50 epithelial cells of three independent experiments. ***p ≤0.001; **p ≤ 0.01; *p≤0.05.

A major upstream signaling molecule of MLCK is calmodulin that is activated by Ca^2+^ [62–65]. To investigate if Ca^2+^ is involved in GC-induced redistribution of active NMII, we determined if GC inoculation would induce Ca^2+^ flux in polarized epithelial cells. We used two Ca^2+^ indicators, FluoForte (Fig 8A–8C) and Fluo-4 (S5 Fig) to determine the cytoplasmic Ca^2+^ level. Polarized T84 (Fig 8A–8C, S5A and S5B Fig) and HEC-1-B (S5C and S5D Fig) were incubated apically with piliated or non-piliated MS11Opa+ or MS11ΔOpa for 4 h. The cells were then loaded with the fluorescent Ca^2+^ indicator, and the cell membrane marked by the membrane dye CellMask. Cells were imaged using 3D-CFM (Fig 8A, S5A and S5C Fig). The mean fluorescence intensity (MFI) of the Ca^2+^ dyes in individual cells was measured to estimate the cytoplasmic level of Ca^2+^ (Fig 8B and 8C, S5B and S5D Fig). Compared to uninoculated cells, polarized T84 cells and HEC-1-B inoculated with either MS11Pil+Opa+ or MS11Pil+ΔOpa exhibited significant increases in the MFI of both FluoForte (Fig 8B) and Fluo-4 (S5B and S5D Fig). The MFIs of both the Ca^2+^ indicators in MS11Pil+ΔOpa-inoculated epithelial cells were significantly higher than those in MS11Pil+Opa+-inoculated cells (Fig 8B, S5B and S5D Fig). However, the MFI of the Ca^2+^ indicator in epithelial cells inoculated with MS11Pil-ΔOpa, was significantly reduced compared to those infected by MS11Pil+ΔOpa, but similar to those infected by MS11Pil+Opa+ (Fig 8B). Treatment with the inhibitor specific for Ca^2+^ release from intracellular storages 2APB or the intracellular Ca^2+^ chelator BAPTA brought the MFI of the Ca^2+^ indicators in both MS11Pil+Opa+ and MS11Pil+ΔOpa-inoculated polarized epithelial cells back to the basal level as seen in uninoculated cells (Fig 8C, S5B and S5D Fig). These results suggest that GC interacting with the apical surface of polarized epithelial cells increases the cytoplasmic level of Ca^2+^, by opening the intracellular Ca^2+^ storages. Opa inhibits and pili may facilitate GC-induced Ca^2+^ flux.

To determine whether GC-induced redistribution of active NMII depends on Ca^2+^, we treated polarized T84 cells with the Ca^2+^ inhibitor 2APB or BAPTA before and during incubation with piliated GC. Both inhibitors decreased the apical: lateral (Fig 8E) and the junction: non-junction FIRs (Fig 8F) of pMLC in both MS11Pil+Opa+- and MS11Pil+ΔOpa-inoculated polarized epithelial cells, as well as the accumulation of pMLC at GC adherent sites (Fig 8D, right panels). The results in this and previous sections together suggest that Ca^2+^-dependent activation of MLCK is responsible for GC-induced accumulation of active NMII at the apical junction and GC adherent sites.

## Discussion

A primary challenge in our understanding of GC pathogenesis in the FRT is to mechanistically explain why a small percentage of GC infections lead to invasive diseases while the rest of the infections remain localized. A major research obstacle is a lack of infection models that mimic the anatomic environment and process of GC infection *in vivo*. This study utilized our newly established human endocervical tissue model with the support of the traditional polarized epithelial cells. Our results demonstrate that GC can penetrate into the subepithelium of the endocervix, and the efficiency of GC penetration is regulated by Opa phase variation. GC enter the subepithelium by disassembling the apical junction and inducing the exfoliation of polarized columnar epithelial cells. These events are caused by the elevation of the cytoplasmic Ca^2+^ level and the activation and reorganization of NMII in epithelial cells. The expression of CEACAM-binding Opa inhibits GC penetration by suppressing NMII activation and redistribution, as well as Ca^2+^ flux, thereby limiting GC-induced junction disruption and exfoliation of polarized endocervical epithelial cells.

Epithelial exfoliation serves as a protective mechanism of the host as the process sheds off host cell-associated pathogens. Muenzner et al. have shown that GC-induced exfoliation of non-polarized human cervical epithelial cells and squamous epithelial cells in the lower genital tract of the female mice reduces GC colonization [44, 45]. In contrast, we show here that the exfoliation of columnar endocervical epithelial cells does not affect GC adherence and invasion, rather it allows for an increase in GC penetration into the subepithelium of the human endocervix. These conflicting results, observed in different infection models, can be explained by differences between the types of epithelial cells and the mechanisms underlying GC- and apoptosis-induced exfoliation. A primary difference between multilayered squamous and monolayered columnar epithelial cells is the number of epithelial cell layers. Multilayered squamous epithelial cells can shed in layers with new layers growing underneath [67], while monolayered columnar epithelial cells exfoliate individual cells to protect the integrity of the epithelial barrier [23, 68, 69]. In line with previous findings, epithelial cells in our human endocervical tissue model shed primarily as individuals, rarely as a layer. Based on these observations, we can reason that individual cell shedding from the monolayered endocervical epithelium has a much less impact on GC colonization than the shedding of epithelial layers from multilayered squamous cells.

The second major difference between squamous and columnar epithelial cells is the cell-cell junction, the former by adherens junctions (E-cadherin-E-cadherin interactions) and focal adhesion (integrin-extracellular matrix interactions) [70–72] and the latter by apical junctions (consisting of tight and adherens junctions) and desmosomes [73, 74]. Different junctional complexes suggest that GC require two distinct mechanisms to regulate exfoliation and infection in the two types of epithelial cells. In support of this hypothesis, GC-induced exfoliation of squamous epithelial cells has been shown to be regulated through CD105-dependent activation of integrin [44, 45]. Here we show that GC induce the exfoliation of the columnar endocervical epithelial cells by disrupting the apical junction through reorganizing its actomyosin support. While integrins may also be involved in the exfoliation of columnar epithelial cells as they mediate the interaction between epithelial cells and the basal membrane, the apical junction plays the essential role in holding and sealing the columnar epithelial monolayer [75]. GC-induced apical junction disruption weakens the barrier function of the epithelium, thereby allowing the penetration of GC into the endocervical subepithelium. In contrast, the exfoliation of squamous epithelial cells may not significantly impact the barrier function of the epithelium due to the presence of additional cell layers. These data together provide explanations for clinical observations that GC rarely cause symptomatic vaginitis [76] but can be found in the subepithelium of the endocervical biopsies from GC-infected women [77].

Whether GC-induced junctional disruption is sufficient to allow for GC to penetrate through the paracellular space of the endocervical epithelium remains a challenging question. We have previously shown that GC-induced junction disruption does not significantly increase the diffusion of soluble fluorescent dyes from the apical to basolateral chambers of well-polarized T84 cells, but it causes an increase in the lateral mobility of the plasma membrane over the apical junction, leading to reductions in cell polarity [51]. Here we confirm that GC-induced junctional disassembly also leads to a decrease in the polarity of endocervical epithelial cells in human tissues, supporting the existence of such an event in human infection.

Our studies demonstrate a causative relationship between apical junction disruption and epithelial exfoliation, as the pharmacological inhibition of Ca^2+^ flux and NMII activation and the natural expression of Opa suppress both GC-induced apical junction disassembly and exfoliation of polarized epithelial cells. This is in sharp contrast to the exfoliation process involved in the columnar epithelial renewal and wound repair, where the apical junction remains intact [23, 68]. While the precise mechanism underlying the normal exfoliation of endocervical epithelial cells has not been examined, previous studies have shown that the barrier function of the epithelial monolayer is maintained by recruitment of apical junctional proteins and actomyosin to the plasma membrane of cells that neighbor the exfoliating cell [23, 25, 68, 78]. Actomyosin-generated forces in neighboring cells probably push exfoliating cells out while junctional proteins maintain the epithelial barrier. However, in GC-infected cells, the junctional proteins ZO1 and E-cadherin are redistributed from the apical junction to the cytoplasm and intracellular vesicles respectively, and NMII is recruited to GC adherent sites and the apical junction, but not to the plasma membrane of cells neighboring exfoliating cells. As over-activation of NMII in perijunctional actomyosin rings can induce the disassembly and internalization of junctional proteins [15], our data suggest that the exfoliation of GC-infected endocervical epithelial cells is induced by GC actively via the reorganization and activation of their NMII, modifying the normal exfoliation process to facilitate GC penetration into the subepithelium.

A significant finding of this study is that NMII is a target of GC to induce the disassembly of the apical junction and the exfoliation of the endocervical epithelial cells. When the activation and redistribution of NMII are inhibited by pharmacological reagents or Opa expression, GC-induced disassembly of the apical junction and exfoliation are reduced. While the essential role of NMII in the apical junction prohibits us from using a knockdown approach, the catalytic site-specific peptide inhibitor of MLCK PIK [54] and the inhibitory effect of naturally expressed Opa have confirmed the results. Our analysis further suggests that GC-induced NMII activation that leads to exfoliation and GC transmigration primarily depends on MLCK- rather than ROCK-mediated phosphorylation of MLC. Even though both MLCK and ROCK contribute to the phosphorylation of MLC, the two differentially regulate the distribution of active NMII, as the ROCK inhibitor further increases while the MLCK inhibitors reduce the level of active NMII in the apical junction. This result suggests that the subcellular location rather than the level of NMII activation is important for GC infection.

Using piliated and non-piliated MS11ΔOpa, we found that pili promote but Opa inhibits GC penetration into the endocervical epithelium and GC-induced columnar epithelial exfoliation by enhancing or suppressing the activation and redistribution of NMII and Ca^2+^ flux. While the promoting effects of pili are associated with an increase in GC adherence, the inhibitory effects of Opa is independent of GC adherence and invasion. Using MS11ΔOpa expressing a single Opa that cannot undergo phase variation, we showed that the inhibitory effects of MS11Opa+ are mediated by CEACAM-binding Opa but not HSPG-binding Opa. These suggest that CEACAM-binding Opa targets to NMII in columnar epithelial cells, different from its target reported in squamous epithelial cells. Thus, the same Opa may use different mechanisms to suppress the exfoliation of polarized endocervical and non-polarized ectocervical epithelial cells. The inhibitory effect of Opa on GC-induced Ca^2+^ flux supports that the cytoplasmic Ca^2+^ is a target of CEACAM-binding Opa proteins to regulate NMII [56]. However, the exact underlying mechanism is unknown. The inhibitory effects of OpaH may be mediated through engaging CEACAMs [79], which function to enhance cell-cell adherence and suppress cell signaling [80, 81]. This study found that MS11ΔOpa induced a significantly higher level of pMLC accumulation in the apical junction in CEACAM-expressing T84 cells than MS11Opa+ (Fig 6E), but increased the pMLC accumulation to a level similar to MS11Opa+ in HEC-1-B cells that do not express CEACAMs [61] (S4 Fig). These results support the hypothesis that CEACAMs are involved in suppressing GC-induced NMII redistribution. While potentially involved in the exfoliation of both squamous and columnar epithelial cells, CEACAMs may differentially modulate signaling induced by GC, due to distinct distributions of CEACAMs and organizations of signaling, cytoskeleton, and cell-cell junctions in the two types of epithelial cells. Our results also show that GC-induced NMII activation depends on Ca^2+^ flux that can activate MLCK via calmodulin [56]. In addition to Ca^2+^ flux, CEACAM-binding Opa proteins can potentially inhibit GC-induced NMII activation in a Ca^2+^-independent manner, such as by activating MLC phosphatase that dephosphorylates MLC [82] or through modulating GC-epithelial physical interactions. By interacting with CEACAMs on epithelial cells and LOS on neighboring GC, Opa potentially alters the physical tensions that GC exert onto the mucosal surface, consequently changing the organization of NMII beneath the plasma membrane of epithelial cells [83, 84]. Since different isoforms of the 11 Opa proteins have different binding abilities to CEACAMs [85], they can modulate GC-epithelial interactions and signaling distinctly when Opa undergoes phase variation.

**Fig 9:**
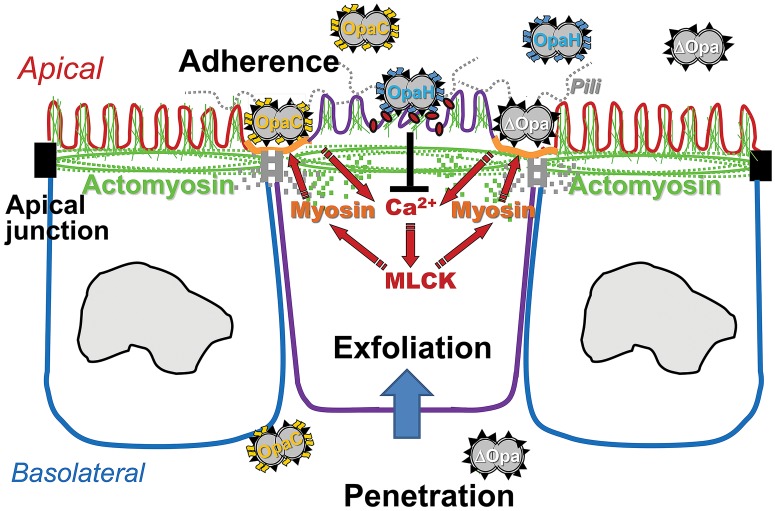
GC modulate infection in the human endocervix by manipulating NMII-mediated epithelial exfoliation through phase variation of Opa and pili. The results presented here suggest that the pili-initiated interactions of GC with the apical surface of the human endocervical epithelial cells induce Ca^2+^- and MLCK-dependent activation and redistribution of NMII to GC adherent sites. NMII activation and remodeling cause the disassembly of the apical junction, which leads to epithelial exfoliation and GC penetration into the endocervical epithelium. The expression of CEACAM-binding OpaH, but not HSPG-binding OpaC, inhibits GC-induced Ca^2+^ flux and NMII activation and redistribution, consequently reducing GC-induced epithelial exfoliation and GC penetration.

This study is the first to utilize human endocervical tissue explants to examine the mechanism by which GC establish infection in this *in vivo* location. Our results have extended our mechanistic understanding of GC pathogenesis in the context of human infection. Based on these results, we propose the follow working model for GC infection in the human endocervix (Fig 9). Pili-initiated interactions of GC with the apical surface of the human endocervical epithelial cells induce Ca^2+^- and MLCK-dependent activation and redistribution of NMII to GC adherent sites. NMII activation and remodeling cause the disassembly of the apical junction that holds the epithelium together and seals the paracellular space. Apical junction disruption leads to epithelial exfoliation and GC penetration into the endocervical epithelium. Phase variation of Opa from HSPG-binding to CEACAM-binding isoforms inhibits GC-induced Ca^2+^ flux and NMII activation and redistribution, consequently reducing GC-induced epithelial exfoliation and GC penetration. The accumulated data from previous and current studies indicate that GC manipulate the epithelial barrier by regulating host cell signaling and cytoskeletal systems for their infection. The nature and level of GC-mediated manipulation are modulated by phase variation of GC surface molecules and types of epithelial cells that GC interact with, which enable GC to infect various regions of the FRT and generate different infection outcomes.

## Materials and methods

### Neisseria strains and epithelial cells

*N*. *gonorrhoeae* strain MS11 that expressed both pili and Opa (MS11Pil+Opa+) was obtained from Dr. Herman Schneider, Walter Reed Army Institute for Research. Derivatives of this strain, MS11ΔOpa, MS11OpaH (CEACAM-binding), and MS11OpaC (HSPG-binding) have previously been described [38, 39]. MS11Opa+ and MS11ΔOpa strains express similar LOS [39]. MS11 Pil+Opa+ and Pil-Opa+ colonies were identified based on their morphology using a dissecting light microscope. Our previous sequencing analysis showed that they expressed different *pilE* variants [38]. GC were grown on plates with GC media (Difco, BD Bioscience, Franklin Lakes, NJ) and 1% Kellogg’s supplement [86] for 16–18 h before inoculation. The concentration of GC in suspension was determined using a spectrophotometer and inoculated at MOI 10:1. Human colorectal carcinoma cell line, T84 (ATCC, Manassas, VA), was maintained in DMEM:Ham F12 (1:1) supplemented with 7% heat-inactivated fetal bovine serum (FBS). Human endometrial adenocarcinoma cell line, HEC-1-B (ATCC), was maintained in Eagles MEM alpha medium supplemented with 10% heat-inactivated FBS. Cells were seeded at 6x10^4^ per transwell (6.5 mm diameter and 3 μm pore size, Corning, Corning, NY) and cultured for ~10 days until transepithelial electrical resistance (TEER) reached >1000 Ω (T84) or >300 Ω (HEC-1-B). TEER was measured using a Millicell ERS volt-ohm meter (EMD Millipore, Billerica, MA).

### Human endocervical tissue explants

The tissue explants were cultured as previously described [87]. Endocervical tissues were obtained from patients undergoing voluntary hysterectomies and received within 24 h post-surgery. Samples were cut into ~2.5 cm (L) X 0.6 cm (W) X 0.3 cm (H) pieces, incubated in CMRL-1066 (GIBCO, Gaithersburg, MD) plus antibiotics for 24 h, and then switched to antibiotic-free media for 24 h.

### Immunofluorescence analysis of human endocervical tissue explants

Individual endocervical tissue pieces were incubated with GC at a MOI of ~10 (based on the average number of endocervical epithelial cells in endocervical tissue pieces) in the presence or absence of the MLCK inhibitor ML-7 (10 μM, EMD Millipore) and PIK (Dreverse,100 μM) [54] on transwells for 24 h. Unassociated GC were removed by extensive washes at 6 and 12 h. The tissue was fixed, embedded in gelatin, cryopreserved, sectioned crossing the apical and basolateral surfaces of the epithelium, stained for ZO1 (BD Bioscience), pMLC (Cell Signaling Technology, Beverly, MA), and GC [51] by specific antibodies, and nuclei by DAPI (Life Technologies, Carlsbad, CA), and analyzed using CFM (Zeiss LSM 710, Carl Zeiss Microscopy LLC, Oberkochen, Germany). Images were acquired as Z-series of 0.37 μm slices, and 3D composites obtained using the NIH ImageJ software.

To quantify epithelial exfoliation, the number of epithelial cells localized on the top of the monolayer (exfoliated) and the total number of epithelial cells were counted by visual inspection in each randomly acquired image, to determine the percentage of exfoliated cells. To estimate the level of GC penetration into the subepithelium, the number of non-exfoliated epithelial cells (in clearly visible epithelial monolayers) with basal GC staining and the total number of GC-associated epithelial cells were visually counted to calculate the percentage of infected epithelial cells with GC penetration into the basolateral side. To evaluate the disruption of the apical junction, the number of GC-associated epithelial cells that lost continuously apical staining of the junctional protein ZO1 and the total number of GC-associated epithelial cells were visually counted to calculate the percentage of GC-associated epithelial cells with apical junction disassembly. To quantify the redistribution of pMLC, the fluorescence intensity ratios (FIR) of pMLC staining at the apical to lateral in individual epithelial cells were determined using average FI as previously described [51].

### Immunofluorescence analysis of polarized epithelial cells

Cells were pretreated with or without NMII kinase inhibitors, Y27632 (10 μM, EMD Millipore), ML-7 (10 μM, EMD Millipore) and PIK (100 μM) or Ca^2+^ inhibitors, 2APB (10 μM, EMD Millipore) and BAPTA (50 μM, EMD Millipore) for 1 h, and incubated with GC in the presence or absence of the inhibitors for 6 h. Cell were washed and fixed with 4% paraformaldehyde, permeabilized with 1% Triton X100, and stained with anti-E-cadherin (BD Bioscience), anti-pMLC (Cell Signaling Technology, Carlsbad, CA), anti-GC antibodies, and DAPI for nuclei. Cells were analyzed by CFM. Images were acquired as Z-series of 0.37 μm slices, and 3D composites obtained. Epithelial exfoliation will be quantified using xz images as described for the tissue explants. The distribution of E-cadherin and pMLC was quantitatively analyzed by measuring the FIR at the apical junctional to the cytoplasmic area (from xy images) or at the apical to lateral surface area (from xz images) in individual cells.

### Fence functional of the apical junction

Polarized T84 cells were incubated with GC apically for 6h. Then the cells were incubated with the CellMask dye (5μg/ml, Life Technologies) in the basolateral chamber only for 15 min, and xz images were acquired using Leica TCS SP5 X confocal microscope (Leica Microsystems, Buffalo Grove, IL). The number of epithelial cells displaying CellMask staining at the apical membrane was countered visually as the percentage of the total number of epithelial cells in each randomly acquired image.

### Immunoblotting analyses

Polarized T84 cells, apically incubated with GC for 6 h with or without inhibitors, were lysed by RIPA buffer [0.1% triton × 100, 0.5% deoxycholate, 0.1% SDS, 50 mM Tris-HCl pH 7.4, 150 mM NaCl, 1 mM EGTA, 2 mM EDTA, 1 mM Na_3_VO_4_, 50 mM NaF, 10 mM Na_2_PO_4_, and proteinase inhibitor cocktail (Sigma-Aldrich, St. Louis, MO)]. Lysates were resolved using SDS-PAGE gels (BioRad, Hercules, CA) and analyzed by Western blot. Blots were stained for pMLC or MLC (Cell Signaling Technology), stripped, and reprobed with anti-β-tubulin antibody (Santa Cruz, Santa Cruz, CA). Blots were quantified using a Fujifilm LAS-3000 (Fujifilm Medical Systems U.S.A., Inc., Stamford, CT).

### GC adherence, invasion, and transmigration assays

The assays were performed as previously described [51]. Briefly, polarized epithelial cells that were pretreated with or without the inhibitors for 1 h were incubated apically with GC at 37°C for 3 h for adherence and 6 h for invasion and transmigration assays with or without the inhibitors. GC in the basolateral media were cultured and counted as transmigrated bacteria. Cells were washed and lysed to count adherent GC. Cells were treated with gentamicin, washed, and lysed to count bacteria that were resistant to gentamicin treatment as invaded GC.

### Calcium imaging

T84 cells were seeded at 1×10^5^ per transwell on the underside of transwells [88] and cultured for ~10 days until TEER reached >1000 Ω. Cells were pre-treated with or without the Ca^2+^ inhibitors, 2APB (10 μM, Sigma, Saint Louis, MO) and BAPTA (50 μM, Sigma), for 1 h and incubated with GC (MOI of 10) apically in the presence or absence of the inhibitors for 4 h. Then cells were incubated with the fluorescent Ca^2+^ indicator Fluoforte (100 μg/ml, Enzo Life Sciences, Farmingdale, NY) or Fluo-4 (100 μM, Life Technologies) for 1 h. Confocal xz images were acquired in the presence of the membrane dye CellMask (5 mg/ml, Life Technology) using Leica TCS SP5X confocal microscope (Leica Microsystems, Buffalo Grove, IL), based on the instruction by manufacturers. To quantify the intracellular Ca^2+^ level, the cytoplasmic region of individual cells was manually selected based on the CellMask staining in randomly acquired confocal images, and the mean fluorescent intensity (MFI) of Fluoforte and Fluo-4 in the cytoplasmic region was measured using the NIH ImageJ software.

### Statistical analysis

Statistical significance was assessed using the Student’s t-test and one-way ANOVA by Prism software (GraphPad Software, La Jolla, CA).

### Ethics statement

Human cervical tissue was obtained from National Disease Research Interchange (NDRI, Philadelphia, PA). Human cervical tissues used were anonymized. The usage of human tissues has been approved by the Institution Review Board of the University of Maryland.

## Supporting information

S1 Fig The effects of inhibitors of Ca^2+^ flux or NMII phosphorylation on GC-induced exfoliation of polarized T84 and the endocervical epithelial cells. (A-B) Polarized T84 cells on transwells were untreated or pre-treated with the ROCK inhibitor Y27632 (Y), the MLCK inhibitors ML-7 (M), and an inhibitor of Ca^2+^ release from intracellular pools, 2APB, for 1 h and apically incubated with MS11Pil+Opa+ for 6 h in the presence or absence of inhibitors. (A) Cells were fixed, stained for DNA and GC, and analyzed using 3D-CFM. Shown are representative images that intercept both the apical and basolateral surfaces (Scale bar, 10 μm). (B) Based on cell nuclear staining, the average percentage (±SD) of exfoliated epithelial cells was determined by counting the number of epithelial cells localizing above the epithelium of T84 monolayers versus the total number of epithelial cells in randomly selected fields. Shown are the results from >15 randomly selected fields (>50 individual cells) from three independent experiments. (C) Human endocervical tissue explants were untreated or pre-treated with PIK (100 μM) for 1 h and incubated with MS11ΔOpa for 24 h in the presence or absence of the inhibitor. Cells were fixed, stained for DNA and GC, and analyzed using 3D-CFM. The average percentages (±SD) of exfoliated cells among the total number of GC-associated epithelial cells were determined from >15 randomly selected fields (>50 cells) of the endocervix of three human subjects. ***p ≤0.001.
(TIF)

S2 Fig Treatment of the NMII motor inhibitor blebbistatin, but not the MLCK inhibitors ML-7 and PIK or the Ca^2+^ inhibitor 2APB induces the exfoliation of polarized T84 cells in the absence of GC. (A) Polarized T84 cells were treated with inhibitors for 6 h, fixed, stained to visualize the cell nuclei, and imaged by 3D-CFM. (B) The percentage of cell moving above the epithelial monolayer (dash lines) was determined from three independent experiments. Scale bar, 5 μm. *p≤0.05.
(TIF)

S3 Fig Ca^2+^, ROCK and MLCK inhibitors have no significant effects on GC growth. MS11Pil+ΔOpa was cultured in DMEM/F12 containing 10% FBS for 24 h in the absence and presence of the ROCK inhibitor Y-27632 (10 μM), the MLCK inhibitor ML-7 (10 μM) or PIK (100 μM), the intracellular Ca^2+^ chelator BAPTA (50 μM), or the Ca^2+^ inhibitor 2APB (10 μM). Optical density at 650 nm was measured at time point 0, 6, 12, and 24 h. Shown are the average OD (±SD) of three independent experiments.
(TIF)

S4 Fig GC interaction increases the levels of phosphorylated MLC at the apical membrane of HEC-1-B cells. Polarized HEC-1-B cells were apically incubated with piliated MS11Opa+ or ΔOpa at a MOI of 10 for 6 h. Cells were fixed, permeabilized, stained for phosphorylated MLC (pMLC) and GC, and analyzed using 3D-CFM. The fluorescence intensity ratio (FIR) of pMLC at the apical to lateral region was determined. Shown are representative xz images (A) and the average FIR (±SD) (B) of >50 individual cells from more than three independent experiments. Arrows indicate GC. Scale bar, 5 μm. *p≤0.05.
(TIF)

S5 Fig GC induce the elevation of the cytoplasmic Ca^2+^ in polarized epithelial cells. Polarized T84 (A and B) and HEC-1-B cells (C and D) were incubated apically with or without piliated MS11Opa+ or ΔOpa (MOI = 10) in the absence or presence of the Ca^2+^ inhibitor 2APB (10 μM) for 4 h. Then, cells were incubated with the Ca^2+^ indicator Fluo-4 and the membrane dye CellMask and analyzed using 3D-CFM. Shown are representative xz images (Scale bar, 5 μm) (A and C) and the average mean fluorescence intensity (MFI) (±SD) of Fluo-4 in the cytoplasmic region (B and D) generated from >50 individual cells of three independent experiments. ***p ≤0.001; **p ≤ 0.01.
(TIF)
