# Supplementary figures and images for: Neisseria gonorrhoeae infects the human endocervix by activating non-muscle myosin II-mediated epithelial exfoliation
Source: PLoS Pathog. 2017 Apr 13;13(4):e1006269. doi: 10.1371/journal.ppat.1006269 (PMC5391109; doi:10.1371/journal.ppat.1006269)

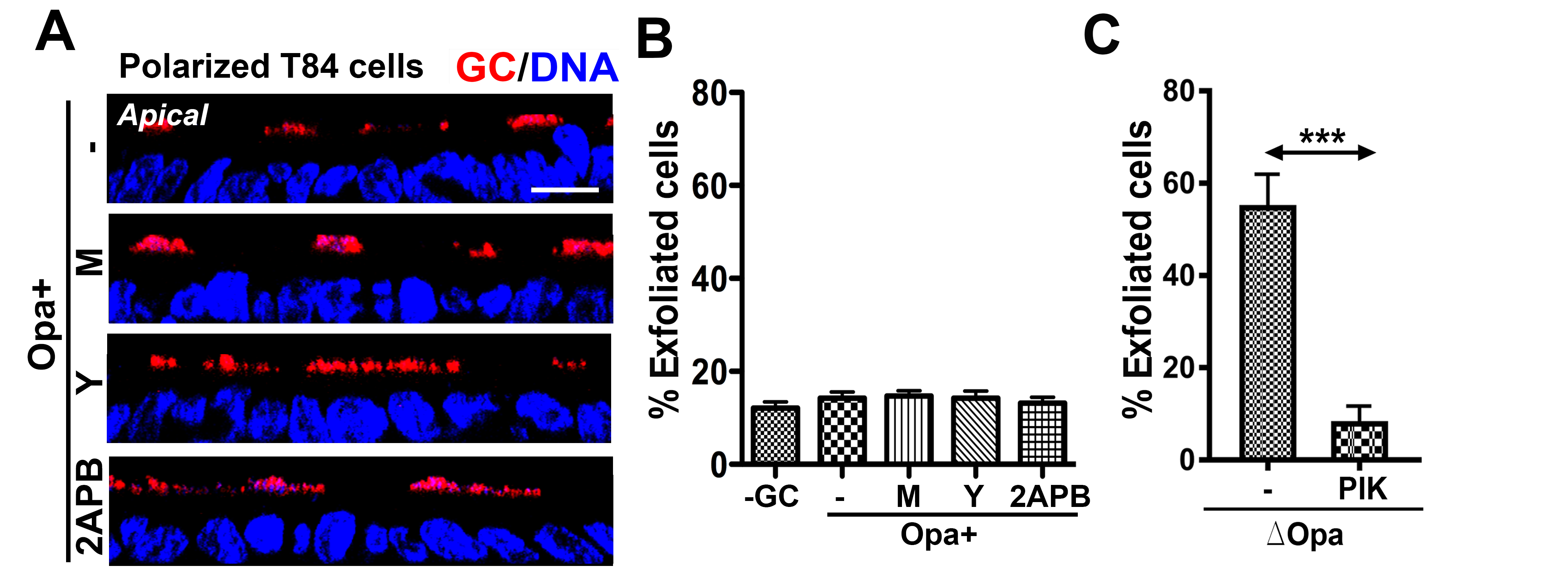

Supplement: S1 Fig — (A-B) Polarized T84 cells on transwells were untreated or pre-treated with the ROCK inhibitor Y27632 (Y), the MLCK inhibitors ML-7 (M), and an inhibitor of Ca2+ release from intracellular pools, 2APB, for 1 h and apically incubated with MS11Pil+Opa+ for 6 h in the presence or absence of inhibitors. (A) Cells were fixed, stained for DNA and GC, and analyzed using 3D-CFM. Shown are representative images that intercept both the apical and basolateral surfaces (Scale bar, 10 μm). (B) Based on cell nuclear staining, the average percentage (±SD) of exfoliated epithelial cells was determined by counting the number of epithelial cells localizing above the epithelium of T84 monolayers versus the total number of epithelial cells in randomly selected fields. Shown are the results from >15 randomly selected fields (>50 individual cells) from three independent experiments. (C) Human endocervical tissue explants were untreated or pre-treated with PIK (100 μM) for 1 h and incubated with MS11ΔOpa for 24 h in the presence or absence of the inhibitor. Cells were fixed, stained for DNA and GC, and analyzed using 3D-CFM. The average percentages (±SD) of exfoliated cells among the total number of GC-associated epithelial cells were determined from >15 randomly selected fields (>50 cells) of the endocervix of three human subjects. ***p ≤0.001. (TIF) [file ppat.1006269.s001.tif]

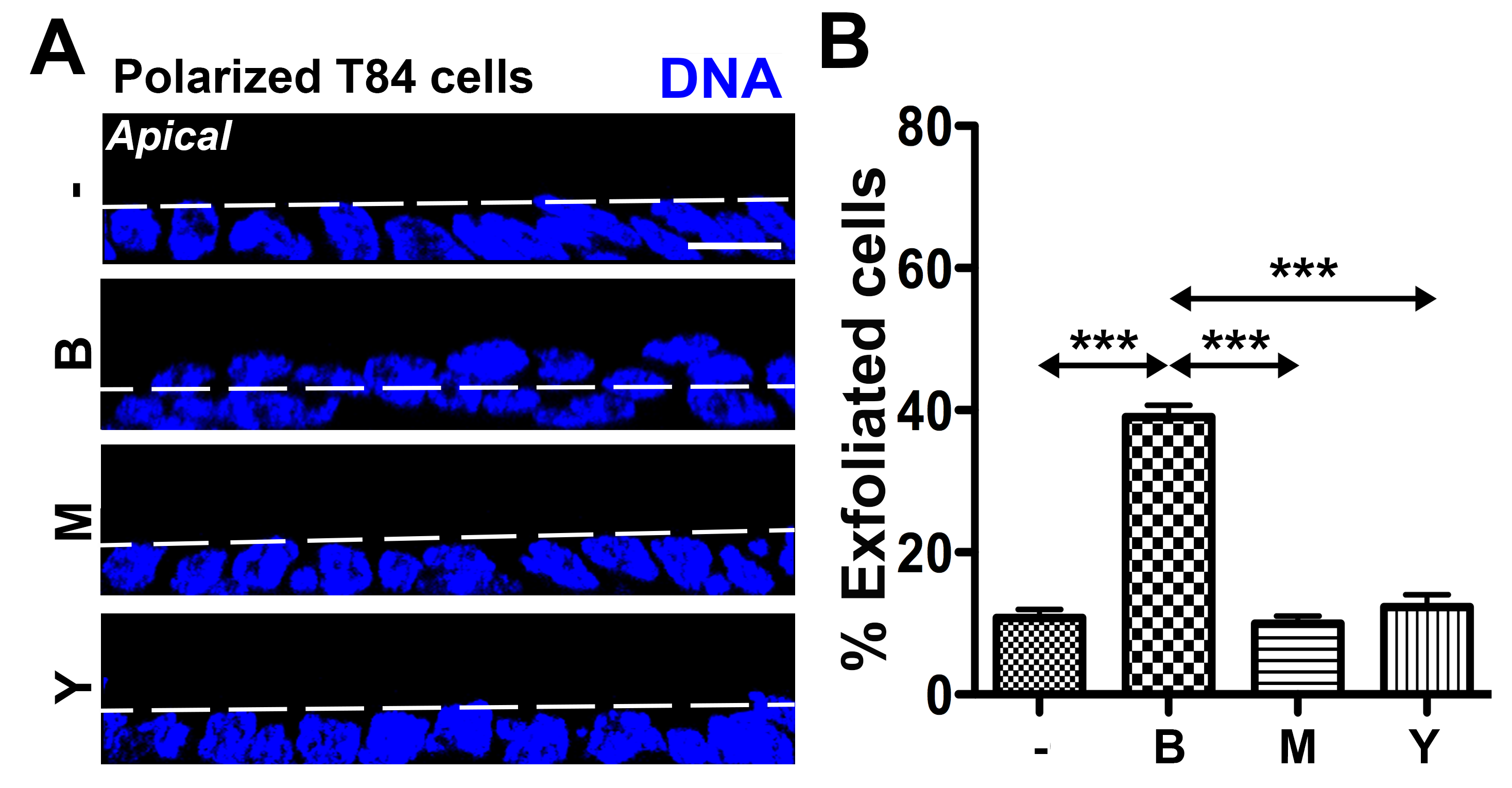

Supplement: S2 Fig — (A) Polarized T84 cells were treated with inhibitors for 6 h, fixed, stained to visualize the cell nuclei, and imaged by 3D-CFM. (B) The percentage of cell moving above the epithelial monolayer (dash lines) was determined from three independent experiments. Scale bar, 5 μm. *p≤0.05. (TIF) [file ppat.1006269.s002.tif]

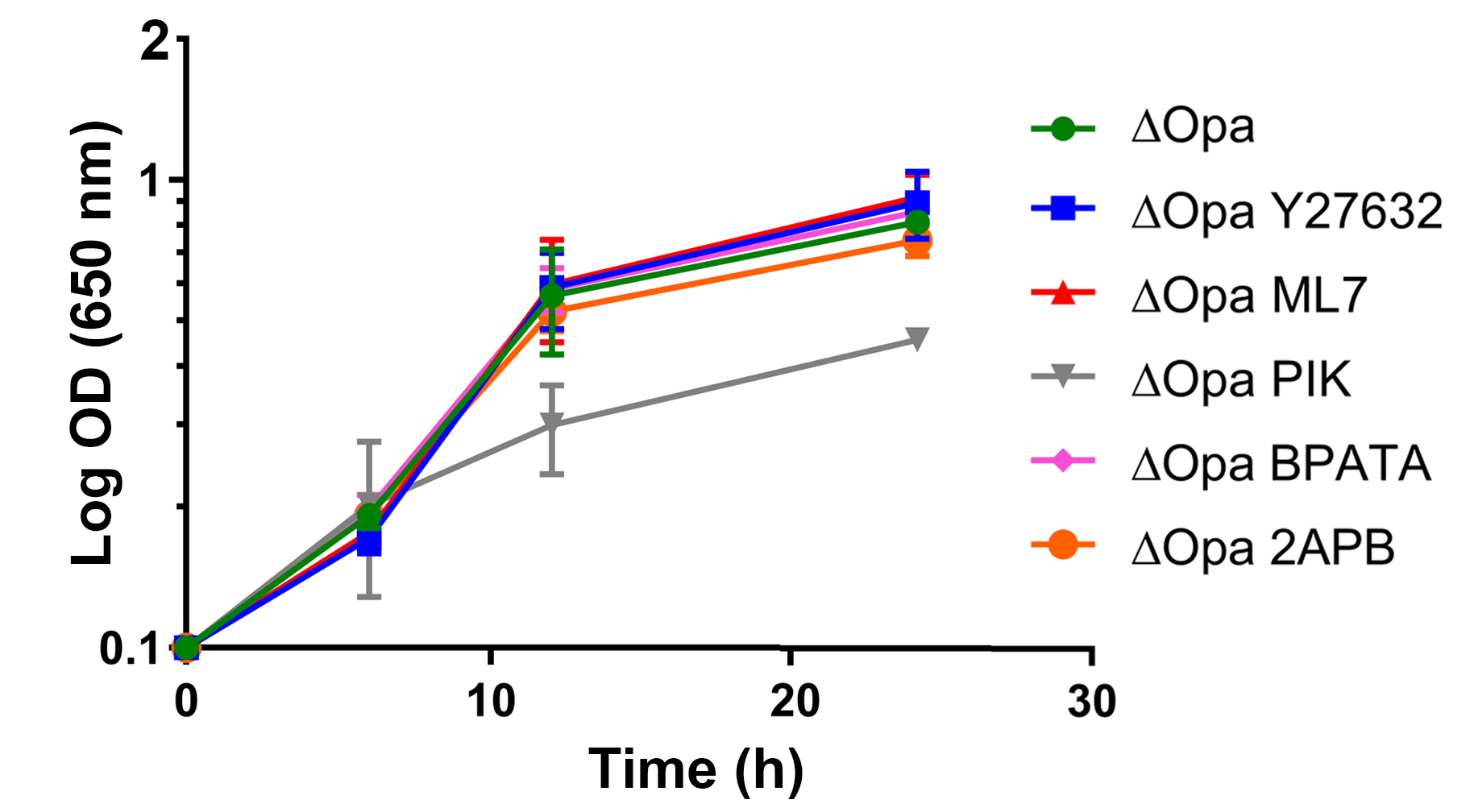

Supplement: S3 Fig — MS11Pil+ΔOpa was cultured in DMEM/F12 containing 10% FBS for 24 h in the absence and presence of the ROCK inhibitor Y-27632 (10 μM), the MLCK inhibitor ML-7 (10 μM) or PIK (100 μM), the intracellular Ca2+ chelator BAPTA (50 μM), or the Ca2+ inhibitor 2APB (10 μM). Optical density at 650 nm was measured at time point 0, 6, 12, and 24 h. Shown are the average OD (±SD) of three independent experiments. (TIF) [file ppat.1006269.s003.tif]

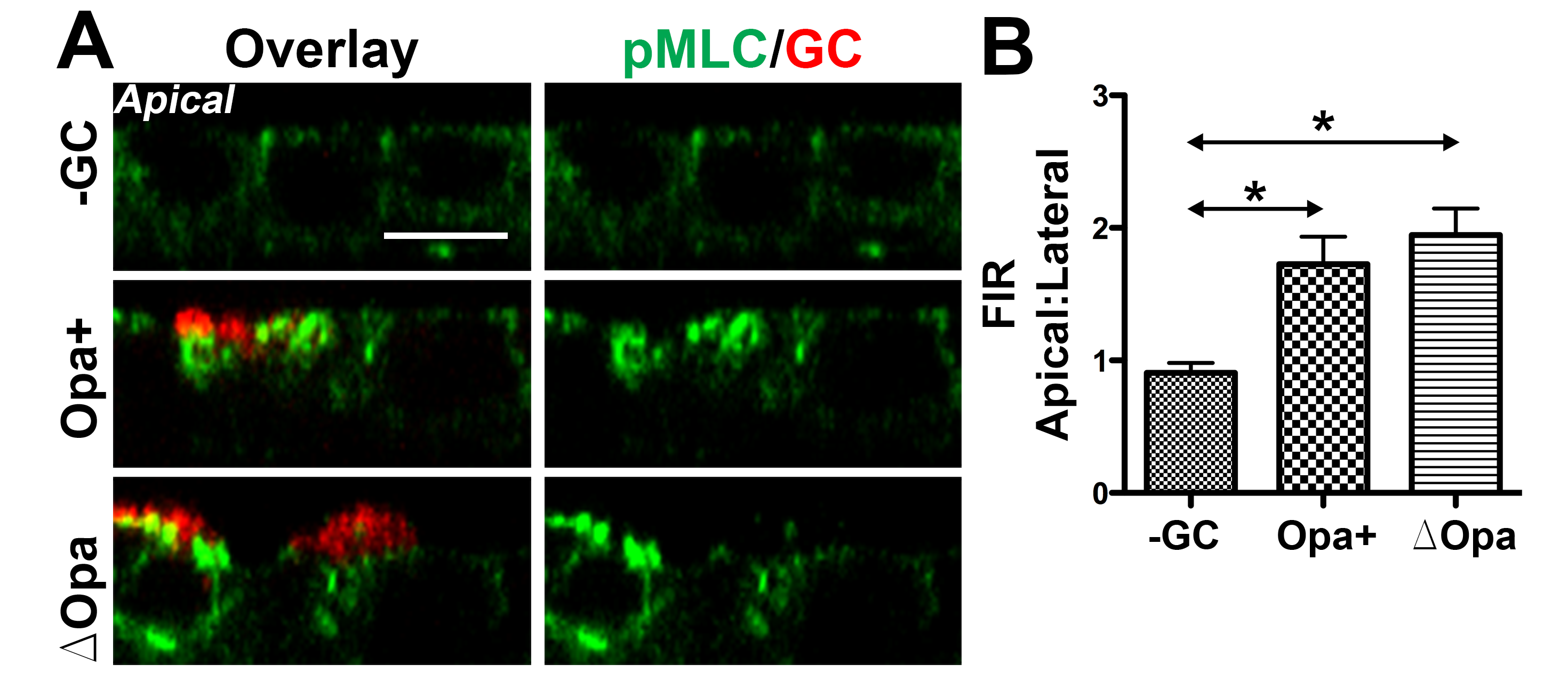

Supplement: S4 Fig — Polarized HEC-1-B cells were apically incubated with piliated MS11Opa+ or ΔOpa at a MOI of 10 for 6 h. Cells were fixed, permeabilized, stained for phosphorylated MLC (pMLC) and GC, and analyzed using 3D-CFM. The fluorescence intensity ratio (FIR) of pMLC at the apical to lateral region was determined. Shown are representative xz images (A) and the average FIR (±SD) (B) of >50 individual cells from more than three independent experiments. Arrows indicate GC. Scale bar, 5 μm. *p≤0.05. (TIF) [file ppat.1006269.s004.tif]

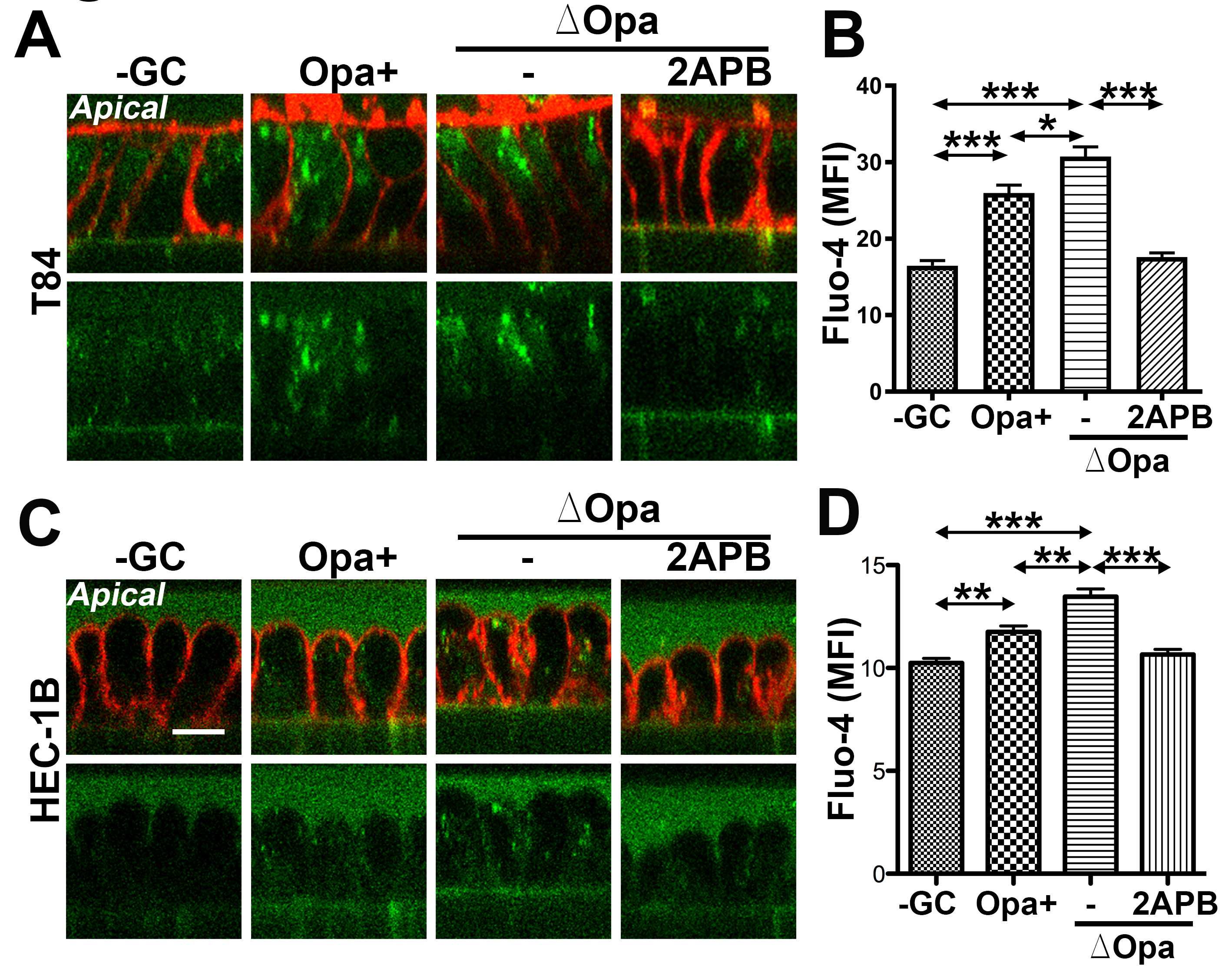

Supplement: S5 Fig — Polarized T84 (A and B) and HEC-1-B cells (C and D) were incubated apically with or without piliated MS11Opa+ or ΔOpa (MOI = 10) in the absence or presence of the Ca2+ inhibitor 2APB (10 μM) for 4 h. Then, cells were incubated with the Ca2+ indicator Fluo-4 and the membrane dye CellMask and analyzed using 3D-CFM. Shown are representative xz images (Scale bar, 5 μm) (A and C) and the average mean fluorescence intensity (MFI) (±SD) of Fluo-4 in the cytoplasmic region (B and D) generated from >50 individual cells of three independent experiments. ***p ≤0.001; **p ≤ 0.01. (TIF) [file ppat.1006269.s005.tif]
